# Genome-wide identification and characterization of the superoxide dismutase gene family in *Musa acuminata* cv. Tianbaojiao (AAA group)

**DOI:** 10.1186/s12864-015-2046-7

**Published:** 2015-10-20

**Authors:** Xin Feng, Zhongxiong Lai, Yuling Lin, Gongti Lai, Conglong Lian

**Affiliations:** Institute of Horticultural Biotechnology, Fujian Agriculture and Forestry University, Fuzhou, Fujian, 350002 China

**Keywords:** *Musa acuminata* cv. Tianbaojiao (AAA group), *SOD* gene family, Promoter, Abiotic stress, Hormonal stress, Expression analysis

## Abstract

**Background:**

Superoxide dismutase (SOD) is an essential enzyme of the plant antioxidant system that responds to oxidative stresses caused by adverse conditions. Banana is an important staple and economic crop in tropical and subtropical regions. However, its growth and yield are constantly affected by various abiotic stresses. To analyze the roles of distinct *SOD* genes under various stresses, a detailed characterization and analysis of the *SOD* gene family in Cavendish banana is indispensable.

**Methods:**

The presence and structure of the SOD family genes were experimentally verified using 5′/3′ RACE-PCR, reverse transcription PCR and PCR. Then, their syntenic relationships, conserved motifs and phylogenetic relationships were analyzed using software. Cis-elements present in the promoters were predicted via PlantCARE. And the expression levels under abiotic and hormonal stresses were determined using real-time quantitative polymerase chain reaction.

**Results:**

In total, 25 ‘Tianbaojiao’ *SOD* cDNAs (*MaSODs*), which encoded six Cu/ZnSODs, four MnSODs and two FeSODs, were cloned. The 12 *MaSOD* genes were divided into four groups based on their conserved motifs, which corroborated their classifications based on gene-structure patterns and subcellular localizations. Eleven *MaSOD* promoters were isolated and found to contain many *cis*-acting elements involved in stress responses. Gene expression analysis showed that 11 out of the 12 *MaSOD*s were expressed in all tested tissues (leaf, pseudostem and root), whereas *MaCSD2B* was expressed only in leaves and roots. Specific *MaSOD* members exhibited different expression patterns under abiotic and hormonal treatments. Among the 12 *MaSOD* genes, *MaCSD1D* was the only one that responded to all eight treatments, suggesting that this gene plays a predominant role in reactive oxygen species scavenging caused by various stresses in banana.

**Conclusions:**

A genome-wide analysis showed that the ‘Tianbaojiao’ banana harbored an expanded *SOD* gene family. Whole genome duplication, segmental duplication and complex transcriptional regulation contributed to the gene expansion and mRNA diversity of the *MaSODs*. The expression patterns of distinct *MaSOD* genes showed that they are important responses to different abiotic and hormonal stresses in banana.

**Electronic supplementary material:**

The online version of this article (doi:10.1186/s12864-015-2046-7) contains supplementary material, which is available to authorized users.

## Background

Banana is an important staple and economic crop in tropical and subtropical regions. However, its growth and yield are constantly affected by severe abiotic and biotic stresses, such as cold in winter, drought and water-logging, as well as various diseases and pests [1]. These environmental perturbations often lead to the increased generation of reactive oxygen species (ROS) in plant cells [2]. Excess ROS can attack virtually all cellular macromolecules. This usually results in membrane damage, protein oxidation and DNA lesions, and can even lead to irreparable metabolic dysfunction and cell death [3, 4]. Thus, to cope with ROS toxicity, plants have developed efficient and complex antioxidative response systems, including many non-enzymatic and enzymatic components. Among these enzymatic components, superoxide dismutases (SODs), acting as the first line of antioxidant systems in plant, play important roles in catalyzing the dismutation of superoxide radicals to protect cells from oxidative damage [5].

In plants, there exist multiple SOD isozymes, which are classified into three types based on their metal co-factors: Cu/ZnSOD, FeSOD and MnSOD [6]. Although these SOD proteins are encoded by nuclear genes, they are distributed to different cellular compartments. Cu/ZnSODs are mainly located in the cytosol, chloroplasts, peroxisomes and/or the extracellular space, while FeSODs are mainly in chloroplasts and possibly the cytosol, and MnSODs are in the mitochondria [7]. Owing to their important roles in the antioxidant system, a considerable number of *SOD* genes are cloned from various monocot and dicot plants [8–12]. However, *Arabidopsis thaliana* and *Populus trichocarpa* are the only two plants whose *SOD* gene families have been characterized in the genome-wide level at present, and the numbers of the three *SOD*-type genes varies among them [8, 9].

*SODs* contribute to various environmental stimuli responses in plants, such as cold, drought, salinity, auxin and ethylene [7, 13, 14]. Different *SOD* genes exhibited different expression patterns. The responses of *SODs* to environmental changes or stresses were dramatically different, depending on the different *SOD* members present, the stress and the species. For instance, under ozone fumigation, the transcriptional levels of chloroplastic *CSD2* and *FSD1* were transiently decreased, while chloroplastic *FSD2* mRNAs remained somewhat constant in *A. thaliana* [8]*.* In contrast, *FSD2* mRNAs dramatically increased in response to UV-B, while *CSD2* or *FSD1* mRNAs remained constant. In addition, cytosolic *CSD1* could be involved in responses to both ozone fumigation and UV-B illumination. Even so, the *SOD* genes of the same *SOD* type did not always exhibit uniform functions in different species. *MnSOD* showed no altered expression when subjected to a series of oxidative stress treatments in *Arabidopsis*, but it responded positively to salt stress in pea [15], and cold and drought stress in wheat [16, 17]. This indicated that the regulation of *SOD* genes is complicated in response to oxidative stress. *Cis*-element and transcription factor analyses of *SOD* promoters from *Arabidopsis* [6], wheat [12] and longan [10] provided some clues on how the *SOD* genes are modulated. Additionally, alternative splicing (AS) and miRNAs have also found to be involved in the regulation of *SOD* expression [18, 19]. Studies using over-expressing or knocked-out plant *SOD* genes have confirmed their functions in improving stress tolerance [20–22].

In previous reports, Zhou et al. studied the SOD isoenzymes in banana using biochemical methods and revealed that cold stress led to the accumulation of different SOD isoenzymes [23]. A quantitative proteomic analysis confirmed the existence of Cu/ZnSOD, MnSOD and FeSOD in banana [24]. However, these studies focused only on the proteins and changes in activity, which were unable to effectively elucidate the exact roles of banana *SODs* under adverse conditions. Recently, the whole-genome sequences of *Musa acuminata* var. DH-Pahang (wild banana, AA group) and *Musa balbisiana* var. Pisang Klutuk Wulang (PKW; wild banana, BB group) were made available to the public [25, 26], facilitating molecular studies on the expression and regulatory mechanisms of banana *SODs* in response to oxidative stress. Using these genomes, we performed a genome-wide identification of the *SOD* gene family in *M. acuminata* cv. Tianbaojiao (Cavendish banana, AAA group) to analyze the transcript types, protein motifs, exon-intron organizations, chromosomal locations and phylogenetic relationships. Then, the putative promoters of banana *SODs* were also isolated and *cis*-elements involved in stress responses were analyzed to further illuminate their transcriptional regulatory mechanisms. Finally, we studied the expression profiles of *MaSOD* family genes under abiotic (cold, heat, drought and salt) and hormonal [abscisic acid (ABA), gibberellin A_3_ (GA_3_), indole-3-acetic acid (IAA) and salicylic acid (SA)] stresses using real-time quantitative polymerase chain reaction (qPCR), which should help determine the functions of each *MaSOD* gene under adverse conditions.

## Methods

### Plant materials and stress treatments

Sterile plantlets of *M. acuminata* cv. Tianbaojiao (Cavendish banana, AAA group) were generated by inducing suckers as Zhang et al. described [27]. Twenty-five-day-old sterile plantlets were used in abiotic stress treatments, except the drought treatment. The plantlets were cultivated in Murashige and Skoog liquid solution with 200 mM NaCl for salt treatment, 100 μM IAA, 100 μM GA_3_ and 100 μM SA for hormonal treatments, and sprayed with 100 μM ABA in 0.02 % (v/v) Tween 20 for the ABA treatment. These treated plantlets were sampled at 4, 8, 12, 24 and 48 h, except that salt treatment, which was sampled at 4, 12, 24, 48 and 72 h. For the drought treatment, 2-month-old plants grown in soil were cultivated without watering and sampled at 1 d, 2 d, 3 d, 4 d and 5 d. All the treatment conditions were at 28 °C with 3300 lux continuous light, except the cold stress, which was conducted in 4 °C growth chambers with 400 lux continuous light, and the heat stress, which was conducted in 40 °C growth chambers with 3300 lux continuous light. Control and treated plantlets were immediately frozen in liquid nitrogen and stored at −80 °C until nucleic acid extraction.

### Nucleic acid extraction and cDNA synthesis

Genomic DNAs were extracted from ‘Tianbaojiao’ banana using the modified CTAB method [28]. Total RNAs were isolated using Column Plant RNA_OUT_ 2.0 Kit (TIANDZ, China). Then, the quality was checked using 1.0 % agarose gel electrophoresis and quantified using spectrophotometry. Total RNAs were reverse transcribed using a Thermo Scientific RevertAid First Strand cDNA Synthesis Kit (Fermentas, EU) for 3’ UTR (untranslated region) and open reading frame (ORF) cloning, and a SMART™ RACE cDNA Amplification kit (Takara, Japan) for 5’ UTR cloning. A GeneRacer™ kit (Invitrogen, USA) was used for the alternative transcriptional start site analysis.

### Isolation of the *SOD* genes from *M. acuminata* cv. Tianbaojiao

“Superoxide dismutase” and published *SOD* sequences in NCBI were used as the index words and probes, respectively, to search for *SOD* genes in the wild banana genome databases (http://banana-genome.cirad.fr/) [29]. Full-length cDNAs of ‘Tianbaojiao’ *SOD* genes were cloned using 5’/3’ RACE-PCR and reverse transcription PCR (RT-PCR), with primers designed from known *SOD* sequences in the NCBI and the banana genome databases. Genomic sequences of *MaSODs* were obtained from DNA templates using PCR. 5’- flanking regions of *MaSOD* genes, 1.0–2.0 kb in length, were generated using PCR with forward primers (designed to regions 2.0 kb upstream of the start codon of ‘DH-Pahang’ *SOD* genes) and reverse primers (designed to regions downstream of the start codon of each *MaSOD*). All of the primer information is listed in Additional files 1 and 2.

PCR reactions were performed according to the instructions of ThermoScientific DreamTaq Green PCR Master Mix (2×) (USA) and Takara LA Taq (Japan). Then, PCR products with acceptable sizes were purified, subcloned into the vector pMD18-T (Takara, Japan), and confirmed by DNA sequencing. The correct sequences of *SOD* genes were abbreviated as *MaCSD*, *MaMSD* and *MaFSD* for *Cu/ZnSOD*, *MnSOD* and *FeSOD*, respectively, with a prefix representing the genus and species.

### Sequence analysis and subcellular localization

Multiple sequence alignments were performed using ClustalX (version 1.83). The molecular weights and isoelectric points of the MaSODs were calculated using the ExPASy ProtParam tool [30]. Syntenic relationships of *SOD* genes were searched in the Plant Genome Duplication Database (PGDD, http://chibba.agtec.uga.edu/duplication/) [31]. Conserved motifs were detected using MEME with the default settings, except that the maximum number of motifs was defined as nine, and the minimum and maximum motif width was set to 20 and 150, respectively. Exon-intron organizational analyses were carried out using the Gene Structure Display Server (http://gsds2.cbi.pku.edu.cn/). Transcriptional response elements of the promoters were predicted using the PlantCARE tool [32].

Subcellular localizations and putative transit peptides were predicted by SoftBerry (http://linux1.softberry.com/), ChloroP1.1 (http://www.cbs.dtu.dk/services/ChloroP/) [33] and MITOPROT (http://ihg.gsf.de/ihg/mitoprot.html) [34]. The coding regions of *MaCSD1D* were amplified using specific primers. The forward primer was 5’-CATG**CCATGG**ATGGTTAAGGCTGTAGCTGTG-3’ and the reverse primer was 5’-GG**ACTAGT**CTCCTGAAGCCCAATGACAC-3’ (restriction enzyme sites are in bold). The amplicons were then ligated into the N-terminus of the green fluorescent protein (GFP) sequence of the pCAMBIA1302 vector to generate the pCAMBIA1302-35S::*MaCSD1D*-*GFP*::NOS construct. The recombinant plasmid was introduced into onion epidermal cells by *Agrobacterium-*mediated transformation according to a reported method [35]. Two days later, the subcellular localization of the *MaCSD1D* protein was detected using an A1R/A1 laser confocal scanning microscope (Nikon, Japan).

### Chromosomal locations and phylogenetic analysis

*MaSOD* genes were mapped to the chromosomes by performing a BLASTn search against the banana genome databases. Unrooted phylogenetic trees based on *SOD* protein sequences were constructed using the maximum likelihood method of the MEGA 5.02 software with the Poisson model and a bootstrap of 1000 replicates [36].

### Real-time quantitative PCR and data analysis

Total RNAs from different tissues (leaf, pseudostem and root) and stress-treated leaves were reverse transcribed with PrimeScript™ RT Master Mix (Perfect Real Time) kit (Takara, Japan) into cDNAs for qPCR analysis. Primers, specific to every *MaSOD* gene, were designed based on the specific 3’ UTR sequences, except that of *MaFSD1B*, which were designed on the 5’ end sequences. A clathrin adaptor complexes medium gene (*MaCAC*) was used as an internal control because of its relatively stable expression level [37]. The specificity of primers was tested and verified by analyzing the melting curve. A gene expression analysis was performed using a LightCycler480 Real-time PCR detection instrument (Roche, Switzerland) and SYBR® Premix Ex Taq™ II (Tli RNaseH Plus; Takara, Japan) with three biological replicates and technical replicates. PCR reactions included an initial denaturation at 95 °C for 3 min, followed by 40 cycles at 95 °C for 10 s, 57 °C for 20 s, and 72 °C for 30 s. Relative expression levels were determined using the 2^-∆∆Ct^ method. Then, the expression patterns of the 12 *MaSOD* genes were clustered by MeV (version 4.8) using the average linkage hierarchical clustering method. The details of the specific primers for qPCR are listed in Additional file 3.

## Results

### Identification of the *SOD* gene family in *M. acuminata* cv. Tianbaojiao

In the wild banana genome databases, 15 sequences in ‘DH-Pahang’ (AA group) and 14 sequences in ‘PKW’ (BB group) were identified *in silico* as *SOD* genes based on annotations and a BLASTn search using known *SOD* genes from NCBI. Excluding the remnant sequences and chimeric genes, there were 13 potentially functional *SOD* genes in ‘DH-Pahang’ and 11 genes in ‘PKW’ (Additional file 4). Two of these genes (GSMU_Achr10T27190_001 and GSMU_Achr10T27220_001 of ‘DH-Pahang’, and ITC1587_Bchr10_T31275 and ITC1587_Bchr10_T31280 of ‘PKW’) were tandemly located on chromosome 10 and shared similar ORF and gDNA sequences, which indicates that they were recently tandemly duplicated. Sequence analysis revealed that the *SOD* ORF lengths and sequence identities of the two wild bananas were somewhat different (Additional file 4). In this study, the presence and the structure of the *SOD* family genes were experimentally verified in the triploid cultivated banana ‘Tianbaojiao’.

A total of 25 different *SOD* cDNAs with intact ORFs were obtained from banana ‘Tianbaojiao’ using 5’ and 3’ RACE-PCR and confirmed by RT-PCR. Two of the cDNAs were described previously [38] and renamed here as *MaCSD1A-1* (GenBank: JQ411718) and *MaCSD1A-2* (GenBank: JQ411719) for the sake of consistency. A sequence alignment and BLAST results using known *SODs*/SODs in the NCBI database as queries revealed that these 25 transcripts were transcribed from 12 different *MaSOD* genes as a result of alternative transcription start sites (ATSSs), AS and alternative polyadenylation (APA). These transcripts, which encoded the same SOD protein, shared an identical ORF but differed in their UTRs. The major *MaSOD* features are summarized in Table 1.

**Table 1:** Characteristics of the *SOD* genes from *M.acuminata* cv. Tianbaojiao

Gene name	cDNA				Protein			gDNA	
	Accession NO.	5’UTR,bp	3’UTR^a^,bp	ORF,bp	Length,aa	Molecular weight,kDa	pI	Length^b^,bp	Accession NO.
*MaCSD1A-1*	JQ411718	11	210	483	160	16.4	6.82	2891	KM017525
*MaCSD1A-2*	JQ411719	14	205	483	160	16.3	6.85	2863	KM017524
*MaCSD1B-1*	JX948788	134	208	459	152	15.2	5.47	2854	KM017526
*MaCSD1B-2*	KM017523	82	-	459	152	15.2	5.47	2854	KM017526
*MaCSD1B-3*	KM017516	79	-	459	152	15.2	5.47	2854	KM017526
*MaCSD1C*	JX948789	66	184	459	152	15.0	5.82	2341	KM017514
*MaCSD1D*	KC007552	65	187	459	152	15.5	4.87	1807	KM017512
*MaCSD2A-1*	JX519461	82	227	684	227	23.1	6.11	4651	KM017515
*MaCSD2A-2*	KM017517	22	167	684	227	23.1	6.11	4651	KM017515
*MaCSD2B*	KJ739805	45	276	675	224	22.7	7.16	3887	KM017513
*MaMSD1A-1*	JX519462	27	194	717	238	26.3	7.09	4447	KM017530
*MaMSD1A-2*	KM017528	-	156	717	238	26.3	7.09	4447	KM017530
*MaMSD1A-3*	KM017536	-	141	717	238	26.3	7.09	4447	KM017530
*MaMSD1A-4*	KM017529	-	116	717	238	26.3	7.09	4447	KM017530
*MaMSD1B-1*	JQ364939	65	177	732	243	26.5	6.75	3030	KM017531
*MaMSD1B-2*	KM017534	-	156	732	243	26.5	6.75	3030	KM017531
*MaMSD1B-3*	KM017535	-	112	732	243	26.5	6.75	3030	KM017531
*MaMSD1C-1*	JX844024	25	233	738	245	26.8	7.90	4720	KM017532
*MaMSD1C-2*	KM017527	-	213	738	245	26.8	7.90	-	-
*MaMSD1D*	KJ739806	44	223	732	243	26.4	6.76	3121	KM017533
*MaFSD1A-1*	JX535809	26	245	906	301	34.2	5.66	4080	KM017518
*MaFSD1A-2*	KM017520	-	223	906	301	34.2	5.66	4080	KM017518
*MaFSD1A-3*	KM017521	-	189	906	301	34.2	5.66	4080	KM017518
*MaFSD1B*	KJ739807	70	453	783	260	29.9	6.75	4208	KM017519
*MaFSD1B*-*variant1*	KM017522	-	-	687	228	26.2	7.01	3853	KM017519

^a^The length of 3’UTR doesn’t contain polyA

^b^The gDNA is corresponding to the ORF of *MaSODs*

**Fig. 1:**
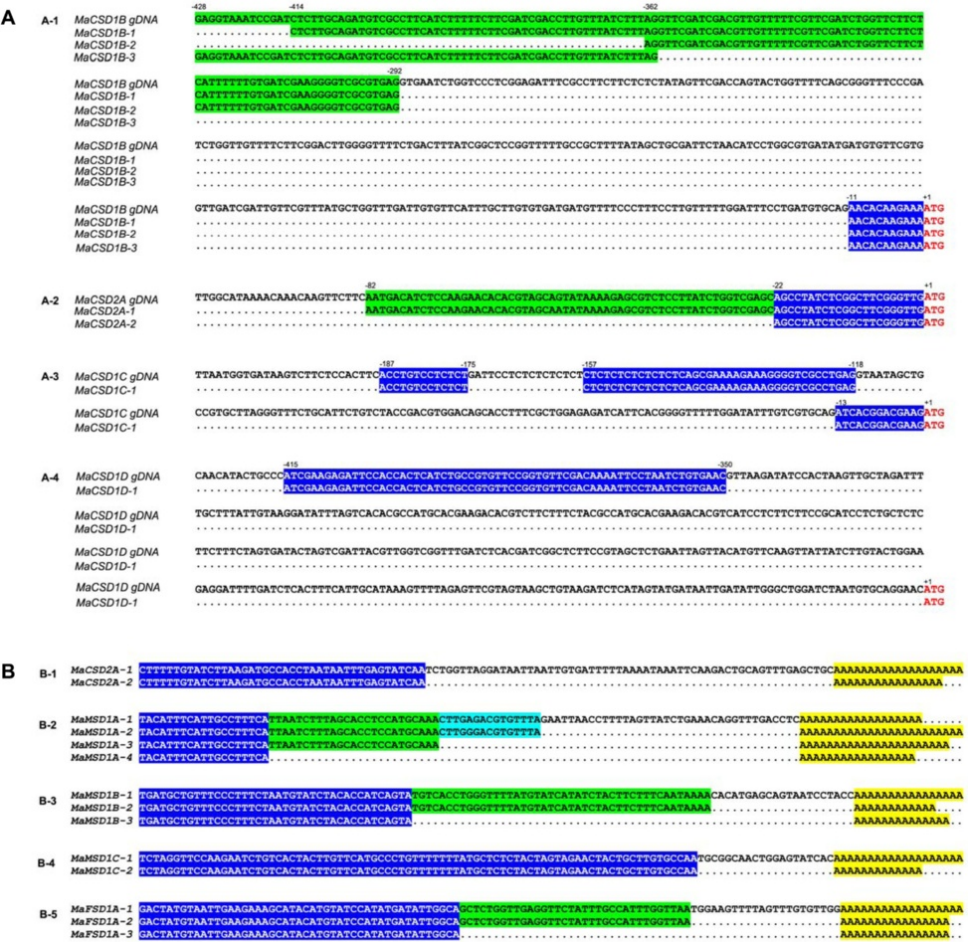
Alignment of 5’-and 3’-ends of *MaSOD* cDNAs. **a** Alignment of 5’-ends of *MaSOD* cDNAs and their corresponding gDNAs. Coordinates are nucleotide positions relative to the translational start site (ATG). **b** Alignment of 3’-ends of *MaSOD* cDNAs

**Fig. 2:**
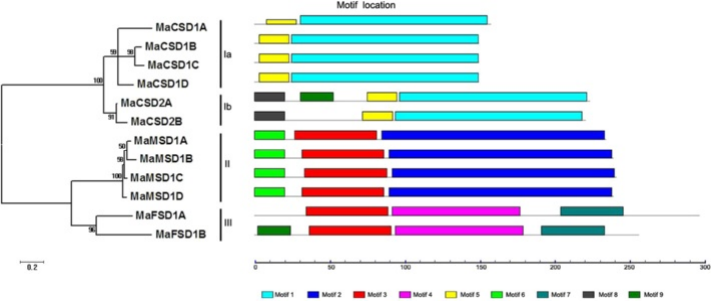
Conserved motifs and clustering analysis of MaSOD proteins. Clustering tree resulting from the amino acid alignments of MaSODs is shown on the left of the figure. The distribution of conserved motifs among the deduced MaSOD proteins is shown on the right of the figure. Different motif types are marked by different color blocks as indicated at the bottom of the figure. Group Ia and Ib are copper/zinc superoxide dismutases, Group II are manganese superoxide dismutases, and Group III are iron superoxide dismutases

The ORF of *MaCSD1A-1* was 98.14 % identical to that of *MaCSD1A-2*. A further analysis of their corresponding gDNAs (GenBank: KM017525 and KM017524) revealed they were alleles. Three independent transcripts encoding MaCSD1B, named *MaCSD1B-1,-2* and −*3*, shared the same 459 bp ORF but varied in the 5’ UTR length or nucleotide composition owing to AS and ATSSs (Fig. 1a-1). Two other transcripts contained a 459-bp ORF but encoded two different amino acid polypeptides, which were named as *MaCSD1C* and *MaCSD1D*. The two transcripts, designated as *MaCSD2A-1* and *MaCSD2A-2*, contained the same ORF of 684 bp but with different 3’ and 5’ UTRs (Fig. 1a-2 and b-1). The 996-bp long cDNA *MaCSD2B* had a 675-bp ORF with a 45-bp 5’ UTR and a 276-bp 3’ UTR.

**Fig. 3:**
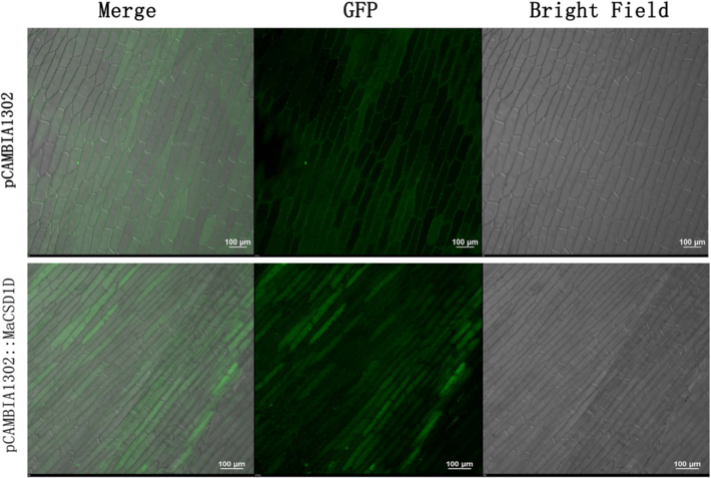
Subcellular localization analysis of MaCSD1D. The upper panel presents the merged fluorescence, fluorescence and bright filed image of GFP control. The lower panel presents the merged fluorescence, fluorescence and bright filed image of MaCSD1D-GFP

**Fig. 4:**
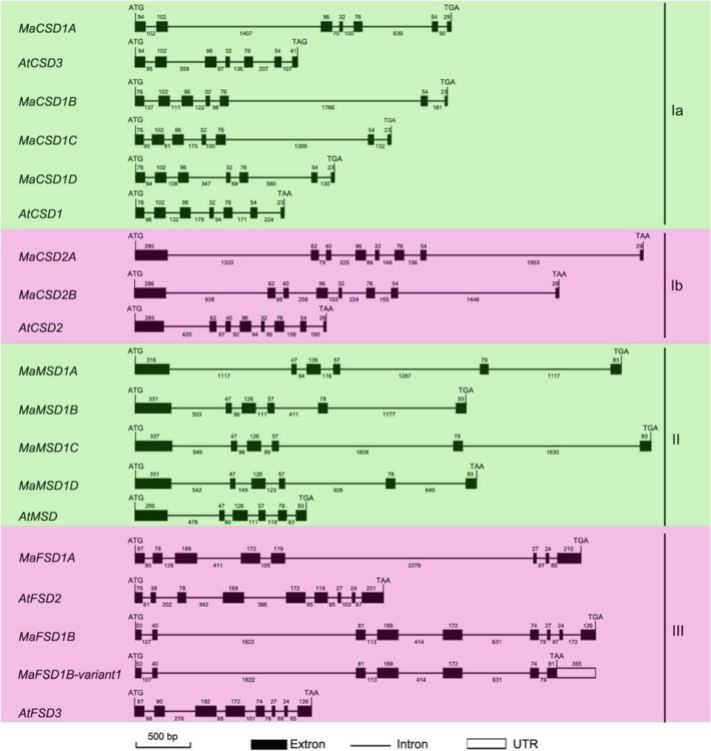
Gene structures of *MaSODs* and *AtSODs*. Exons are shown as black boxes, introns are shown as thin lines, and UTRs are shown as white boxes

Ten independent transcripts of *MnSOD* were classified into four *MaMSD* genes (named *MaMSD1A*, *1B*, *1C* and *1D*) based on the sequence differences of their ORFs and deduced amino acids. Each *MaMSD* gene contained two to four types of transcripts with different 3’ UTR lengths (Fig. 1b-2, b-3 and b-4), resulting from APA, except *MaMSD1D*, which had a unique 3’ UTR (Table 1). APA sites were also found in the *MnSOD* genes of *Larix gmelinii* [39].

**Fig. 5:**
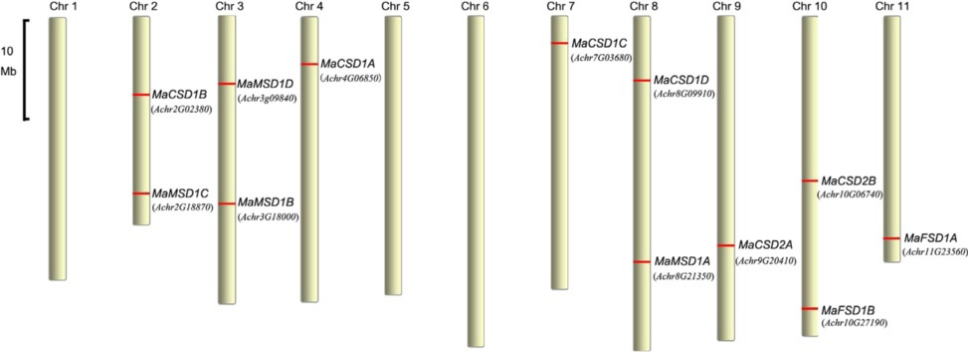
Chromosomal locations of *MaSOD* genes. The positions of *SOD* genes are marked with red lines on the chromosomes. Locus names of ‘DH-Pahang’ *SOD*s are in brackets; for simplicity, the prefix “GSMU_” and suffix “_001” are omitted from each gene name

Multiple sequence alignments of the five *MaFSD* transcripts showed that they encoded two *FeSOD* genes with distinct ORFs. *MaFSD1A-1*,*-2* and −*3*, co-encoded a 301-amino acid polypeptide, contained the same 906 bp ORF but had different 3’ UTR lengths (Fig. 1b-5). The other two cDNAs shared 100 % identity, except that one of them contained two extra sequences of 87 bp and 172 bp in the ORF region (Additional file 5). Aligning with gDNA sequences showed that these two extra sequences were introns retained in the mRNA during splicing, forming an alternative splicing transcript named *MaFSD1B-variant1*. Comparing *MaFSD1B-variant1* with the normal transcript *MaFSD1B* revealed that its ORF was shorter with a premature stop codon.

In addition, the isoelectric points of the 12 MaSOD proteins ranged from 4.87 to 7.90, and their molecular weights varied from 15.0 to 34.2 kDa (Table 1). Pairwise similarities among the 12 *MaSOD* genes were performed and are listed in Additional file 6. The analysis revealed that the *MaMSD* genes shared 80.9–86.5 % identity at both the nucleotide and deduced amino acid levels, followed by *MaCSD* genes at 39.7–87.5 % and *MaFSD* genes at 33.3–42.5 %, whereas the similarity between genes from different *MaSOD* types, such as *MaCSD1A* and *MaMSD1A*, was below 35.6 %. A BLASTp search of the NCBI database revealed that the putative polypeptides of the *MaSOD* genes shared about 62.0–89.0 % sequence identity with orthologous SOD proteins in other plants.

Intragenome syntenic relationship analysis indicated that *MaCSD2A* and *2B*, and *MaMSD1A* and *1C* or *1D* are derived from banana whole genome duplications (WGDs). Cross-genome syntenic analysis revealed that another five *MaSOD* genes (*MaCSD1A*, *MaCSD1D*, *MaMSD1B*, *MaFSD1A* and *MaFSD1B*) are contained in blocks shared among different plant species, suggesting that the other duplicated copies of them in banana genome are lost after WGDs.

### Conserved motifs and clustering analysis of MaSODs

To elucidate the domain features and phylogenetic relationship of the MaSODs, an unrooted phylogenetic tree and a linear distribution map of the conserved motifs in the deduced MaSODs were generated (Fig. 2). MaSODs were clustered into three major clades, which showed a good accordance with their metal cofactor types. The three clades were designated as groups I, II and III. Group I contained two subgroups (Ia and Ib) and harbored motif 1 and motif 5, which contained Cu/ZnSOD signatures and conserved Cu^2+^ and Zn^2+^ binding sites (Additional file 7). Compared with Group Ia, Group Ib included an additional motif 8, which contained chloroplast peptide signal sequences, suggesting MaCSD2A and 2B were located in chloroplasts, whereas MaCSD1A-1D were cytosolic Cu/ZnSODs as predicted by SoftBerry. The MaCSD1D protein fused with GFP to co-express on onion epidermal cells, confirming the cytosolic localization (Fig. 3). Four MaMSDs formed group II, which contained the motifs of 2, 3 and 6. Motif 2 included the conserved metal-binding domain “DVWEHAYY” and five residues (Gly, Gly, Phe, Gln and Asp) [9] that were present in MnSOD but absent from FeSOD, while motif 6 included mitochondrial location signal sequences, suggesting they targeted to the mitochondria. Group III was made up of MaFSD1A and MaFSD1B, which shared motifs 3, 4 and 7. Residues (Ala, Gln, Trp, Phe and Ser) responsible for recognizing iron ion and active sites for FeSOD [40] were found in motif 4 and the conserved metal-binding domain “DVWEHAYY” was detected in motif 7.

### Gene structures of *MaSODs*

The gDNA lengths of *MaSODs* from the start to stop codons varied between 1807 and 4720 bp (Table 1). A gene-structure map was produced by aligning ORF gDNA sequences with their corresponding cDNA sequences (Fig. 4). Interestingly, the *MaSOD* gene structures could also be divided into four groups (Ia, Ib, II and III) based on their exon numbers, which is in agreement with the motif-based classification. *MaSODs* in the same group contained an equal number of exons, with one exception (group III). The four *MaCSD1s* in group Ia possessed seven exons. However, the size of the first exon in *MaCSD1A* was the same as the one in *AtCSD3*, while the size of the first exon in the other three *MaCSD1s* (*MaCSD1B*, *1C* and *1D*) was the same as that in *AtCSD1*. In group Ib, both *MaCSD2A* and *MaCSD2B* had eight exons, just like *AtCSD2*. Similar to previous reports of *AtCSDs* and *PtCSDs* [9], the size of the second exon in *MaCSD1s* (102 bp) corresponded to that of the second and third exons in *MaCSD2s* (62 + 40 bp). This indicates that the inferred exon merge (or split) was shared by monocot and dicot species and should thus to be a defining characteristic of angiosperms. The four *MaMSD* genes belonging to group II contained six exons. In contrast to the conserved genomic structures of groups Ia, Ib and II, the genes in group III exhibited different exon-intron organizational patterns. *MaFSD1A* contained eight exons (one less than its ortholog *AtFSD2*), while *MaFSD1B* possessed nine (one more than its ortholog *AtFSD3*) and *MaFSD1B*-*variant1* harbored seven.

Additionally, many of the introns in banana *SOD* genes were longer than those in their *Arabidopsis* homologs, resulting in longer *MaSOD* gDNAs (Fig. 4). All *MaSOD* introns were spliced in accordance with the eukaryotic GU-AG splice junction site rules [41] with two exceptions: the fourth intron of *MaCSD2A* and the second of *MaMSD1D*, which had splice boundaries of GC-AG. Additionally, one intron was found in the 5’ UTRs of *MaCSD1B* and *MaCSD1D* gDNAs, while two were found in the 5’ UTR of *MaCSD1C* gDNA (Fig. 1a-1, a-3 and a-4).

### Chromosomal locations and phylogenetic analysis

The chromosomal locations of *MaSOD* genes were determined by performing a BLASTn search against the banana genome databases. Eight out of the 11 chromosomes harbored *MaSOD* genes (Fig. 5), four (chr 2, 3, 8 and 10) of which possessed two *MaSOD* genes, while the others (chr 4, 7, 9 and 11) contained only one.

**Fig. 6:**
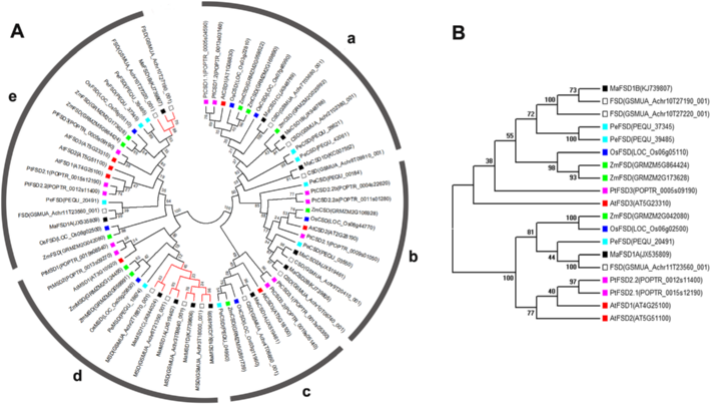
Phylogenetic trees of the *SOD* genes from banana and other plants. **a** A maximum likelihood-based phylogeneitc tree of *SOD* families. GenBank accession numbers or locus IDs are in brackets. a–e refer to different gene clusters. **b** A phylogeneitc tree limited to the FeSODs in group e

To investigate the phylogenetic relationships of MaSODs with other plant SODs, an unrooted phylogenetic tree was constructed from an aligned dataset of MaSODs and 58 homologous SODs from *M. acuminata* var. DH-Pahang, *Zea mays*, *Oryza sativa*, *Phalaenopsis equestris*, *A. thaliana*, and *P. trichocarpa*. The two major clades of SODs were well supported in this tree (Fig. 6a). All Cu/ZnSODs formed a large clade comprising three subgroups (groups a, b and c). FeSODs were clustered with MnSODs into another large clade, indicating, as previous reports [6], that these two subgroups (groups d and e) originated from a common ancestor. MaSODs were found in all five subgroups, where they were clustered with ‘DH-Pahang’ SODs with strong bootstrap support; this close relationship is consistent with their high sequence identities (Additional file 4) and implies that they originated from the same gene.

MaCSD1B, 1C and 1D grouped with AtCSD1 and other cytosolic Cu/ZnSODs in group a, while MaCSD1A was placed into another subgroup along with AtCSD3 (group c). The position of these *MaCSD1s* is consistent with the size of their first exons (Fig. 4), suggesting that two copies of *CSD1* existed before the monocot/dicot split, with only one undergoing expansion in banana (to *MaCSD1B*, *1C* and *1D*). MaCSD2A and MaCSD2B clustered in group b with other plant chloroplastic Cu/ZnSODs.

Interestingly, all four MaMSDs, like PtMSDs and ZmMSDs, were found on a species-specific cluster of the MnSOD clade; this placement indicates that MnSOD gene number amplifications were mainly due to recent WGDs or segmental duplications [9, 31]. Two MaFSDs (MaFSD1A and 1B) fell into different subgroups in group e, only one of which was well supported (85 % bootstrap support). To confirm this result, a small phylogenetic tree limited to FeSODs of group e was constructed (Fig. 6b). In this tree, each MaFSD was separately grouped with FeSOD members from all considered species, revealing the existence of two ancestral *FeSOD* genes before the monocot/dicot split.

### Isolation and bioinformatics analysis of putative *MaSOD* promoters

**Fig. 7:**
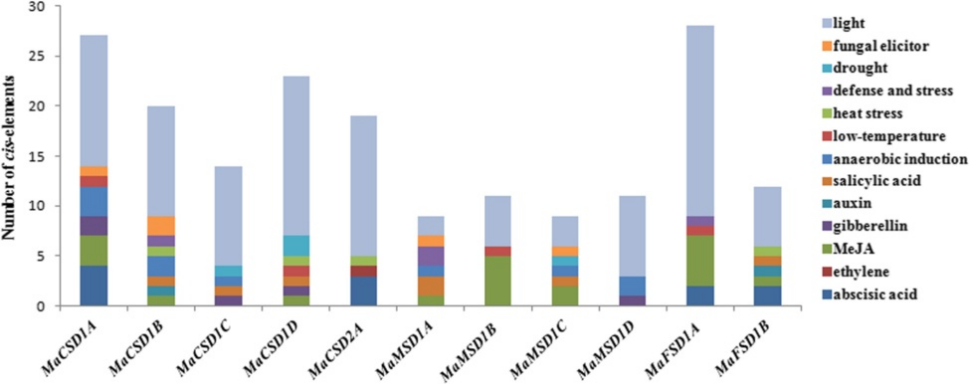
*Cis*-element analysis of putative *MaSOD* promoters related to stress responses. Different *cis*-elements with the same or similar functions are shown in the same color

To further understand and determine the regulatory roles of *MaSODs* under various stresses, the regions upstream of the start codons were isolated by PCR. We obtained a length that varied from 1084 to 2114 bp of 5’-flanking sequences for all *MaSOD* genes except for *MaCSD2B* (Additional file 2). Potential regulatory *cis*-elements that were related to stress and light responses are predicted and summarized in Fig. 7. All 11 putative *MaSOD* promoters possessed typical TATA and CAAT boxes, which are the core elements of the promoters.

As shown in Fig. 7, light-responsive elements accounted for the majority of elements in every *MaSOD* promoter, excluding *MaMSD1A*, *MaMSD1B* and *MaMSD1C*. There were 25 different types of light-responsive elements present in the 11 banana *SOD* promoters. Every promoter possessed two to 10 types, which indicated that *MaSODs* might be differentially regulated when subjected to light. Ten kinds of hormone-responsive regulatory elements were found in the *MaSOD* promoters, such as ABRE, ERE, CGTAC-motif, TGACG-motif, GARE-motif, P-box, TATC-box, AuxRR-core, TGA-box and TCA element, which were associated with ABA, ethylene, methyl jasmonate (MeJA), GA, auxin and SA responses, respectively. Moreover, six types of stress-responsive regulatory elements, TCA elements, AREs, LTRs, HSEs, TC-rich repeats, MBSs and Box-W1s, with responses to anaerobic induction, low-temperature, heat stress, defense and stresses, drought inducibility and fungal elicitors, respectively, were identified in the *MaSOD* promoters. Different types and numbers of regulatory elements were present in distinct *MaSOD* promoters, indicating that *MaSOD* genes should have different regulatory mechanisms in response to various stress and hormone treatments.

### Expression pattern of *MaSOD* genes in different tissues

**Fig. 8:**
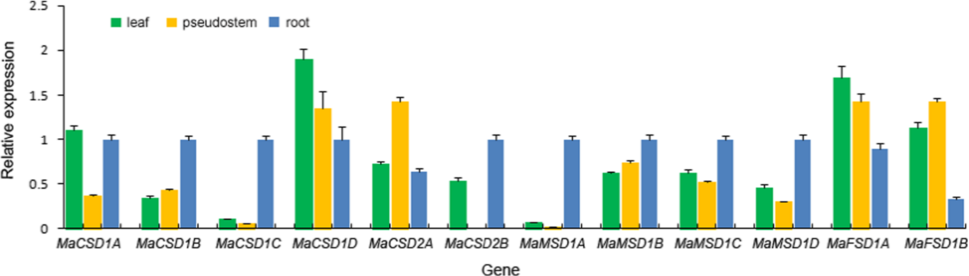
qPCR analysis of *MaSOD* genes in different tissues. Gene names are shown on the x-axis and the expression levels on the y-axis. Different tissues are shown in different colors

qRT-PCR analysis was performed to assess the expression pattern of *MaSOD* genes in different organs of ‘Tianbaojiao’ (Fig. 8). Eleven of the 12 *MaSOD* genes were expressed in all tested tissues (leaf, pseudostem and root), whereas *MaCSD2B* was expressed only in leaf and root tissues. Different *SOD*-type genes in banana had similar expression patterns. *MaCSD1D* and *MaFSD1A* exhibited the highest expression levels in leaves, followed by pseudostems and roots. *MaCSD2A* and *MaFSD1B* were expressed strongly in pseudostems, moderately in leaves and weakly in roots. At mRNA level, *MaCSD1B* and *MaMSD1B* showed their maximum expression in roots, followed by pseudostems and leaves. Expression levels of *MaCSD1C* and *MaMSD1A* were low in leaves and pseudostems and, high in roots.

### Differential expression of *MaSOD* genes in response to various abiotic stresses

**Fig. 9:**
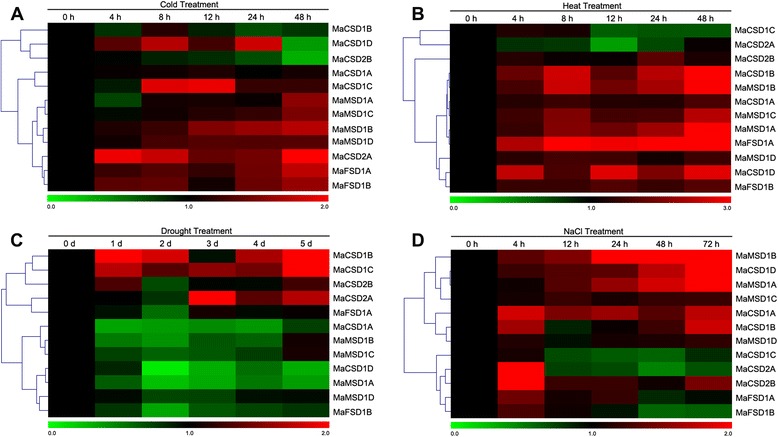
qPCR analysis of differential gene expression under abiotic treatments. The colors of the bar vary from green to red representing the scale of relative expression levels. Each column represents a sampling time point, and each row represents a *MaSOD* member. The expression level of the control (at 0 h/d; marked in black) in every treatment for each gene was used as the rescaled value when calculating the relative expression levels. The clustering results of gene expression patterns are shown on left. **a**–**d** refer to different abiotic treatments

The expression patterns of the 12 *MaSOD* genes as seen by qPCR were detected under cold, heat, drought and NaCl treatments. The results showed that their expression modes were complex (Fig. 9). During cold treatment, the expression levels of most *MaSOD* genes exhibited slight variations. However, there were still four *MaCSD* genes showing obvious differential expression in response to cold stress (*MaCSD2A* and *MaCSD1C* were up-regulated, while *MaCSD1D* and *MaCSD2B* were dramatically down-regulated; Fig. 9a). The heat-treatment-responsive genes were found in all three *SOD*-types. Most of the *MaSOD* genes (*MaCSD1B*, *MaCSD1D*, *MaMSD1A*, *MaMSD1B*, *MaMSD1C* and *MaFSD1A*) were increased at the transcriptional levels, except *MaCSD2A*, which was down-regulated at 12 h (Fig. 9b). Obviously, most *MaSOD* genes were down-regulated in response to drought stress (Fig. 9c). Among them, five members (*MaCSD1A*, *MaCSD1D*, *MaMSD1A*, *MaMSD1B* and *MaFSD1B*) exhibited more than 2- to 10- fold decreases. In contrast, three *Cu/ZnSOD* genes, *MaCSD1B*, *MaCSD1C* and *MaCSD2A*, were strongly induced at 1 d, 5 d and 3 d, respectively. Under the NaCl treatment (Fig. 9d), *MaCSD1D, MaMSD1A* and *MaMSD1B* shared similar expression patterns, which increased gradually to high levels as the treatment continued. The expression of *MaCSD1A* and *MaCSD1B* was dynamic, increasing quickly at the 4-h point, then decreasing gradually, but finally increasing again. In addition, *MaCSD1C*, *MaCSD2A*, *MaCSD2B* and *MaFSD1B* were first up-regulated at 4 h, and then down-regulated over the duration of the treatment.

### Differential expression of *MaSOD* genes in response to hormonal treatments

**Fig. 10:**
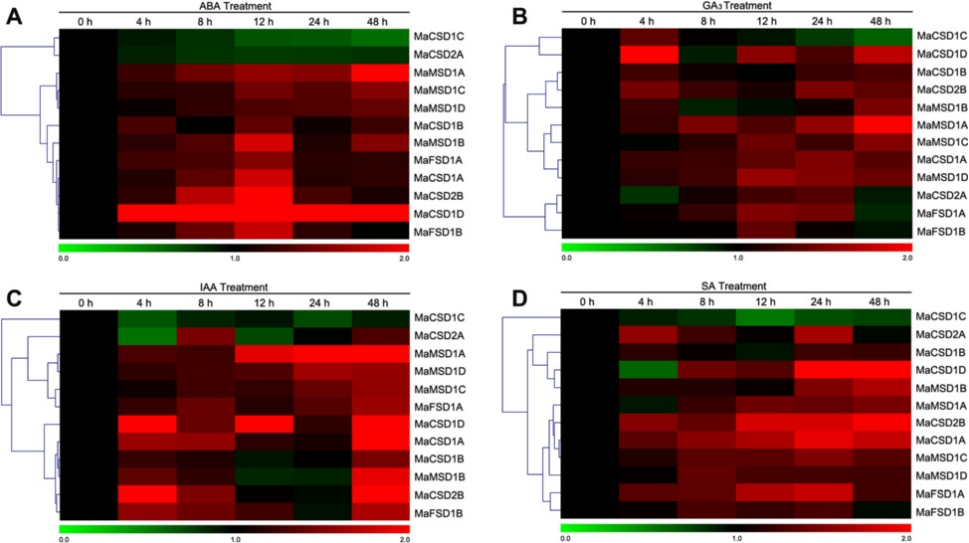
qPCR analysis of differential gene expression under hormonal treatments. The colors of the bar vary from green to red representing the scale of relative expression levels. Each column represents a sampling time point, and each row represents a *MaSOD* member. The expression level of the control (at 0 h/d; marked in black) in every treatment for each gene was used as the rescaled value when calculating the relative expression levels. The clustering results of gene expression patterns are shown on left. **a**–**d** refer to different hormonal treatments

The expression of *MaSOD* genes in response to hormonal stimuli (ABA, GA_3_, IAA and SA) was investigated to further analyze the possible functions of *MaSODs* involved in phytohormone signaling pathways. Most *MaSOD* genes showed an up-regulation in response to hormonal treatments (Fig. 10). Ten out of the 12 *MaSOD* genes were up-regulated to different degrees, whereas the other two (*MaCSD1C* and *MaCSD2A*) were down-regulated under ABA treatment (Fig. 10a). Among the up-regulated genes, *MaCSD1D* exhibited a continuously high-level of transcript abundance over the 48 h time-course with an 8.9-fold peak at 12 h. During the GA_3_ treatment, only two genes (*MaCSD1D* and *MaMSD1A*) were strongly induced at 4 h and 48 h, respectively, while the other genes were slightly induced or repressed with no significant changes (Fig. 10b). The IAA treatment dramatically induced three *MaCSDs* and one *MaMSD* (*MaCSD1A*, *MaCSD1D*, *MaCSD2B* and *MaMSD1A*), but didn’t obviously repress any member (Fig. 10c). Likewise, the expression levels of most *MaSOD* genes were up-regulated in response to the SA treatment, but only *MaCSD1D* was greatly transcribed after 24 h when treated. In addition, the transcription of *MaCSD1C* remained at a low level without a significant change during the treatment time compared with the control (Fig. 10d).

## Discussion

### Expanded *SOD* family in banana

In this study, we identified 12 *SOD* genes representing three major types of plant *SODs* (*Cu/ZnSOD*, *MnSOD* and *FeSOD*) with 25 distinct transcripts from cultivated banana ‘Tianbaojiao’ (AAA group). In contrast to wild bananas, which possess two tandem *FeSOD* genes on chromosome 10, only one *FeSOD*, as assessed by mRNA levels, was isolated from ‘Tianbaojiao’. Further sequence analysis revealed that the two tandem *FeSOD* genes had different putative promoters (1.5 kb upstream of ATG) even thought their ORF and 3’ UTR sequences were similar. Forward primers specific to the two promoters and the same reverse primer (FSD1B-ORFR; Additional file 1) were used to amplify the two tandem *FeSODs* in ‘Tianbaojiao’. PCR amplification of each *FeSOD* using 10 forward primers at different starting positions still yielded only one sequence; this sequence, corresponding to GSMA_Achr10T27190_001 or ITC1587_Bchr10_T31275, was that of the *MaFSD1B* gene. Because the two tandem *FeSOD* genes are present in both the AA and BB genome of *Musa*, the tandem duplication likely predates the A and B genome independent evolution. We propose that the other *FeSOD* on chromosome 10 (corresponding to GSMA_Achr10T27220_001 or ITC1587_Bchr10_T31280) was lost in ‘Tianbaojiao’ over the course of evolution. *MaSOD* genes had more similar ORF lengths and higher sequence identities with their homologs in the AA genome than in the BB genome (Additional file 4). This observation provides additional evidence to support crosses within diploid *M. acuminata* as the source of the edible triploid *M. acuminate* [42].

The number of *SOD* genes varies among monocots (Additional file 8). For instance, the number of distinct *SOD* genes that have been *in silico*-identified ranges from 6 in *Brachypodium distachyon* (3 *Cu/ZnSODs*, 1 *MnSOD* and 2 *FeSODs*) and *Hordeum vulgare* (3 *Cu/ZnSODs*, 1 *MnSOD* and 2 *FeSODs*) to 7 in *O. sativa* (4 *Cu/ZnSODs*, 1 *MnSOD* and 2 *FeSODs*), *Sorghum bicolor* (4 *Cu/ZnSODs*, 1 *MnSOD* and 2 *FeSODs*) and *Setaria italica* (4 *Cu/ZnSODs*, 1 *MnSOD* and 2 *FeSODs*), 9 in *P. equestris* (5 *Cu/ZnSODs*, 1 *MnSOD* and 3 *FeSODs*), 10 in *Z. mays* (5 *Cu/ZnSODs*, 2 *MnSODs* and 3 *FeSODs*) and 18 in *Triticum aestivum* (10 *Cu/ZnSODs*, 2 *MnSODs* and 6 *FeSODs*). Excluding the *SOD* family of hexaploid bread wheat, banana harbors the largest monocot *SOD* gene family, especially with respect to *MnSODs* (four in total). The three whole-genome duplication rounds that have occurred during banana evolution are a major factor responsible for the amplification in gene numbers [25]. However, only five *MaSOD* members (*MaCSD2A* and *2B*, and *MaMSD1A* and *1C* or *1D*) are retained in pairs after banana WGDs, while another five genes (*MaCSD1A*, *MaCSD1D*, *MaMSD1B*, *MaFSD1A* and *MaFSD1B*) are singletons with the loss of other duplicated copies after WGDs. *MaCSD1B*, *1C* and *1D* but not *MaCSD1A* clustered together in Group a (Fig. 6a) and had identical-sized first exons (Fig. 4) and high sequence similarities (Additional file 6); this indicates that *MaCSD1B* and *1C* were probably derived from recent segmental duplications and shared the same origin with *MaCSD1D*.

More than one kind of transcript was obtained from seven of the *SOD* genes in the ‘Tianbaojiao’ banana. Genes, such as *MaCSD2A*, *MaMSD1A*, *MaMSD1B*, *MaMSD1C* and *MaFSD1A*, transcribe two to four different mRNAs as a result of APA. This is not surprising because APA transcripts have already been detected in *Cu/ZnSOD*, *MnSOD* and *FeSOD* genes from *Dimocarpus longan*, *L. gmelinii*, *T. aestivum* and *Z. mays* [10, 12, 43]*.* Like APA, AS is a regulatory mechanism to generate multiple transcripts from one single gene. In this study, two kinds of AS occurred in banana *SODs*. In one, the introns were retained in the 5’ UTR region of *MaCSD1B* mRNA when transcribed, resulting in a normal ORF but a longer 5’ UTR. In the other one, the introns resided in the coding region of *MaFSD1B*, resulting in a premature stop codon, producing a truncated protein (228 aa). Similar observations were made in other plant *Cu/ZnSOD* and *FeSOD* genes [10, 18, 44]. A previous report in rice suggested that introns that resided in the 5’ UTR region were linked to regulating spatially and temporally gene expression [45]. In addition, the rice *FeSOD* variant with a short ORF is sensitive to low temperatures and its truncated protein is active [46], indicating that a similar variant of the *MaFSD1B* gene may play a crucial role in the responses to some abiotic stresses. In spite of these, ATSS also provides a regulatory method to generate multiple transcripts. Although a previous study showed that each type of longan *SOD* genes has multiple transcriptional start site [10], we found only two *MaSOD* genes (*MaCSD1B* and *MaCSD2A*) have ATSSs.

In addition to the above findings, allelic *SOD* genes (*MaCSD1A-1* and *MaCSD1A-2, MaMSD1C-1 and MaMSD1C-2*) with single nucleotide substitutions were detected in the triploid banana ‘Tianbaojiao’. This discovery demonstrates that allelic variation exists inside its three A genomes, which is apparently another mechanism promoting RNA and protein diversity in heterozygotes. Furthermore, the sequence polymorphism found between the *SOD* genes of ‘DH-Pahang’ and ‘Tianbaojiao’ (Additional file 4) is direct evidence of sequence variability in the A genomes.

Taken together, our data provide more detailed sequence information and transcriptional regulatory types for banana *SODs*. Whole genome duplication, segmental duplication and the complex regulation of transcription have contributed to the gene expansion and mRNA diversity of banana *SODs*.

### Specific *MaSOD* members are involved in different abiotic stress responses

Three types of plant *SOD*s (*Cu/ZnSOD*, *MnSOD* and *FeSOD*) are all reported to be in involved in ROS scavenging caused by abiotic stress [8, 16]. The banana *Cu/ZnSOD* subfamily comprises six members (*MaCSD1A-1D* and *MaCSD2A*-*2B*). An expression analysis revealed that every *MaCSD* gene was responsive to at least one abiotic stress treatment performed in this study (cold, heat, drought or salinity) (Fig. 9). Notably, *MaCSD1D* is the only member that showed obvious expression changes under all four abiotic stresses. Compared with other *MaCSD* promoters, the *MaCSD1D* promoter harbored more kinds and numbers of *cis*-elements involved in abiotic stresses, including one LTR motif (*cis*-element involved in low-temperature responsiveness), one HSE motif (*cis*-element involved in heat stress responsiveness) and two MBS motifs (MYB binding site involved in drought-inducibility), which could explain why *MaCSD1D* showed obvious responses to the four abiotic treatments. This indicates that *MaCSD1D* might play a predominant antioxidant role in banana. Similarly, various responses to the abiotic stress were also identified in other plant *Cu/ZnSOD* subfamiles [47].

Banana harbors four *MnSOD* genes (*MaMSD1A-1D*) with high sequences similarities (80.9-86.5 %), which were in accord with their phylogenitic clustering results. Interestingly, individual *MaMSD* genes have some distinguishing features from each other, especially in the 5’ and 3’ UTR regions, indicating that they have evolved over the time and probably underwent modifications to form regulatory diversification under constantly changing environments. This was confirmed by their having different *cis*-elements involved in stress responses. For instance, two TC-rich repeat motifs (*cis*-elements involved in defense and stress responsiveness) were present in the *MaMSD1A* promoter but absent from *MaMSD1B*, *1C* and *1D*. A qPCR analysis showed that *MaMSD1A* was responsive to heat, drought and salt stress, while *MaMSD1B* was responsive to heat and drought stress. *MaMSD1C* was only responsive to heat stress, and *MaMSD1D* was not responsive to any stress. This suggested that the four *MnSOD* genes in banana play distinct roles in scavenging ROS caused by different stimuli.

Additionally, *MaSOD* members located in the same subcellular department also exhibited different expression patterns. The expression of chloroplastic *MaCSD2B* decreased during the cold treatment while that of *MaCSD2A* increased, and vice versa during the NaCl treatment. Likewise, drought up-regulated the expression of cytosolic *MaCSD1B* and *MaCSD1C*, whereas it down-regulated *MaCSD1A* and *MaCSD1D. MaMSD1A* and *MaMSD1B* exhibited increased expression levels under NaCl stress while the other two members (*MaMSD1C* and *MaMSD1D*) showed little change in expression. Our data also show that heat stress represses the expression of chloroplastic *MaCSD2A* but strongly induces chloroplastic *MaFSD1A*. In transgenic tobacco, the over-expression of chloroplastic *FeSOD* suppressed the expression of both chloroplastic and cytosolic Cu/ZnSODs, and the authors proposed that chloroplastic *FeSOD* over-expression interfered with a signal pathway regulating the *Cu/ZnSODs* through a low superoxide radical concentration [48]. Therefore, we hypothesize that there may exist some inter and cross-family signal pathways regulating the expression of *MaSODs* under various environmental stresses.

In addition, the promoters of *MaCSDs* and *MaFSDs* contain large amounts of light responsive *cis*-elements of 5 to 10 different types (Fig. 7). Kurepa et al. detected an increased accumulation of both *Cu/ZnSOD* and *FeSOD* transcripts in tobacco when exposed to light stress [49]. Similar observations were also detected in *Arabidopsis* and rice [7]. Tobacco plants harboring an over-expressed *Cu/ZnSOD* gene from pea exhibited an increased tolerance against high light [50]. Therefore, we propose that *MaCSDs* and *MaFSDs* might participate in light responses.

### *MaSODs* probably participate in phytohormone signaling pathways

Hormone-responsive transcription factors are known to act via combination with their corresponding *cis*-elements in the promoter to regulate the expression of target genes during various stresses [51]. The PlantCARE database predicted that four *MaSOD* promoters (*MaCSD1A*, *MaCSD2A*, *MaFSD1A*, *MaFSD1B*) contained two to four ABREs, a *cis*-acting element involved in the abscisic acid responsiveness, indicating that these genes probably participate in ABA responses. The mRNA levels of *MaCSD1A*, *MaFSD1A* and *MaFSD1B* were induced to 1.8-fold, 1.5-fold and 1.8-fold, respectively, at 12 h under ABA treatment, whereas the transcripts of *MaCSD2A* were slightly reduced over the treatment’s time course. Moreover, the expression of three other members (*MaCSD1D*, *MaCSD2B* and *MaMSD1A*), which had no ABREs, showed >2.0-fold expression inductions during the ABA treatment, suggesting that there are other regulation mechanisms responding to ABA. In the recent study, ABA was shown to regulate the expression of miR398 [52], which was negatively correlated with that of its target genes (*Cu/ZnSODs*) [19, 53]. MiR398 was also reported to exist in banana [25], and a sequence analysis found binding sites for miR398 in the mRNAs of *MaCSD2A*, but not in *MaCSD1A* mRNA, which may explain why the *MaCSD2A* was down-regulated under ABA treatment. We therefore proposed that the expression of *MaSOD* genes in response to ABA may be synergistically mediated by ABREs and miRNAs. In addition to ABA, tobacco *SOD* genes were also reported to be responsive to auxin, gibberellin A and other substances [54]. In this study, we predicted using the PlantCARE database that there were eight other hormone-responsive *cis*-elements (ERE, CGTA-motif, TGACG-motif, GARE-motif, P-box, TATC-box, AuxRR-core and TCA-element) located 5’-upstream of *MaSODs*. We confirmed their transcriptional regulation under GA_3_, IAA and SA treatments using qPCR. Furthermore, H_2_O_2_ produced in the dismutation reaction by SODs is also a signal molecular that can interact with phytohormones to affect various metabolic processes in the cell under stress [55–57]. We hypothesize that *MaSODs* probably participate in phytohormone signaling pathways.

## Conclusions

Banana harbors 12 *SOD* genes, including three types of plant *SODs* (*Cu/ZnSOD*, *MnSOD* and *FeSOD*). Whole genome duplication, segmental duplication, APA, AS and ATSS have contributed to the gene expansion and mRNA diversity of banana *SODs*. The 12 *MaSODs* were distributed on eight out of the 11 banana chromosomes. Based on structural characteristics, the 12 MaSODs were divided into four groups. Promoter sequence analyses revealed that there were many abiotic and hormonal-responsive *cis*-elements in the 5’ upstream regions of the *MaSODs*, but distinct members harbored different types and numbers, which suggested that the 12 *MaSODs* were differentially regulated. A qPCR analysis revealed that distinct *MaSOD* genes exhibited different expression patterns in response to abiotic and hormonal stresses, which indicated that specific *MaSOD* members play roles in different aspects of banana abiotic stress tolerance and hormonal signaling pathway.

## Additional files

Additional file 1: Table S1. Oligonucleotide primers used for cloning *MaSOD* genes. (PDF 81 kb) Additional file 2: Table S2. Oligonucleotide primers used for cloning the 5’- flanking regions of *MaSOD* genes. (PDF 102 kb) Additional file 3: Table S3. Specific primers used for quantitative real-time PCR. (PDF 62 kb) Additional file 4: Table S4. Information of *SOD* genes from the wild and cultivated bananas. (PDF 13 kb) Additional file 5: Figure S1. Sequence alignment of *MaFSD1B* and *MaFSD1B*-*variant1. (PDF 211 kb)*
Additional file 6: Table S5. Pairwise identity of the coding region cDNAs and deduced amino acids of *MaSOD* genes. (PDF 13 kb) Additional file 7: Table S6. Motif sequences of *MaSODs* identified by MEME tools. (PDF 14 kb) Additional file 8: Table S7. Potential functional *SOD* family genes identified *in silico* in monocot plants. (PDF 13 kb)

## Abbreviations

SOD: Superoxide dismutase
CSD or Cu/ZnSOD: Copper/zinc superoxide dismutase
MSD or MnSOD: Manganese superoxide dismutase
FSD or FeSOD: Iron superoxide dismutase
APA: Alternative polyadenylation
AS: Alternative splicing
ATSS: Alternative transcription site
ABA: Abscisic acid
GA_3_: Gibberellin A_3_
IAA: Indole-3-acietic acid
SA: Salicylic acid
ROS: Reactive oxygen species
PKW: *Musa balbisiana* var. Pisang Klutuk Wulang
CAC: Clathrin adaptor complexes medium
WGD: Whole genome duplication
