# Supplementary material for: Genome-wide identification and characterization of the superoxide dismutase gene family in Musa acuminata cv. Tianbaojiao (AAA group)
Source: BMC Genomics. 2015 Oct 20;16:823. doi: 10.1186/s12864-015-2046-7 (PMC4615540; doi:10.1186/s12864-015-2046-7)
Supplement: Additional file 1: Table S1. — Oligonucleotide primers used for cloning MaSOD genes. (PDF 81 kb) [file 12864_2015_2046_MOESM1_ESM.pdf]

Additional file 1: Table S1. Oligonucleotide primers used for cloning *MaSOD* genes.

Gene name	Prime sequences （5′ - 3′）	Tm / °C	Extension time / s	Purpose
MaCSD1A	CSD1A-ORFF: ATGGCAACGGGAGGCAATCTG	55.0	30 (for ORF),	ORF/gDNA amplification
	CSD1A-ORFR: TCAAACTGCTGACTGAAGACC		140 (for gDNA)	
	CSD1A-3RACEF1: GGGTACACGCATGTGAGGGGAA	57.0	50	3′ RACE of *MaCSD1A-1*
	CSD1A-3RACEF2: GATGAGGAACGTCATGCGGGTGAC	60.2	25	
	CSD1A-3RACEF1: GGGTACACGCATGTGAGGGGAA	57.0	50	3′ RACE of *MaCSD1A-2*
	CSD1A-3RACEF3: TTATGCGGACCAAATTCGGTTCTG	58.3	25	
	CSD1A-5RACER1: AACCACGACTGCCCTTCCCAAAATTG	58.0	30	5′ RACE of *MaCSD1A-1*
	CSD1A-5RACER2: CTTCCCAGAACCGAATTTGGTCCG	58.0	30	5′ RACE of *MaCSD1A-2*
MaCSD1B	CSD1B-ORFF: CACCACTTCTCGAGTCTTCCTC	57.0	30 (for ORF),	ORF/gDNA amplification
	CSD1B-ORFR: CATTCCCTCCACTCACAGACTAC		140 (for gDNA)	
	CSD1B-3RACEF1: CACGGTGCCCCTGAAGATGACAAT	58.0	30	3′ RACE
	CSD1B-3RACEF2: GAGCAAGTTGTAGAATTCCAGTAGTC	57.0	15	
	CSD1B-5RACER1: TGCCTCTCTTGATTAATTGTGCTGC	56.9	35	5′ RACE
	CSD1B-5RACER2: ACTCACAGACTACTGGAATTCTAC	58.0	18	
MaCSD1C	CSD1C-ORFF: CCTGAGATCACGGACGAAGATGG	57.0	30 (for ORF),	ORF/gDNA amplification
	CSD1C-ORFR: CTCTATGACAAAACCTCAGCATCCAAC		130 (for gDNA)	
	CSD1C-3RACEF1:GACACCACCAATGGTTGCATGTC	56.0	30	3′ RACE
	CSD1C-3RACEF2: GATCTTGGAAAAGGTGGGCATGAGCTG	59.0	18	
	CSD1C-5RACER1:CAGCTCATGCCCACCTTTTCCAAGATC	57.0	30	5′ RACE
MaCSD1D	CSD1D-ORFF: GAACATGGTTAAGGCTGTAGCTG	56.0	30 (for ORF),	ORF/gDNA amplification
	CSD1D-ORFR: GCAACTGTACAAGCAGCTTCATCC		110 (for gDNA)	
	CSD1D-3RACEF1:CCATGTTTGACAGCAAGATCGCAC	57.0	24	3′ RACE
	CSD1D-3RACEF2: GAGTGACTGATCCAAGGGGAATGAT	58.0	12	
	CSD1D-5RACEF1:CACCAGAATTTCCAGTGGTCTTGC	55.9	35	5′ RACE
	CSD1D-5RACEF2: CAGTGCCATCGTCTCCAGCTATC	58.0	27	
MaCSD2A	CSD2A-ORFF: ATGCAAGCCACACTCGCCGC	58.0	40 (for ORF),	ORF/gDNA amplification
	CSD2A-ORFR:GATTGATACTCAAATTACTAGGTGGC		270 (for gDNA)	
	CSD2A-3RACEF1: TCGAGCAGCCTATCTCGGCTTC	57.0	56	3′ RACE
	CSD2A-3RACEF2: CTTTCCCAAGGTCGCCTCCTC	58.5	48	
	CSD2A-5RACER1:CCCAATGAGATGCTAGAAACCATTAGG	57.0	48	5′ RACE
	CSD2A-5RACER2:GATCATCCTCAAGTTCATGAACCAC	58.0	34	
MaCSD2B	CSD2B-ORFF: GTGCCTTATCTGTACGCCGCTC	58.0	40 (for ORF),	ORF/gDNA amplification
	CSD2B-ORFR: CCATGGGAAGGAGACTGCAACTC		220 (for gDNA)	
	CSD2B-3RACEF1: CCTCTTCCACCGCCAAGCTC	57.0	54	3′ RACE
	CSD2B-3RACEF2: CAAGCTGCTTCTTTCACAGACGG	58.0	15	
	CSD2B-5RACER1: GACAACAGGCTTATTAGGTGGCATC	57.0	60	5′ RACE
	CSD2B-5RACER2: GATCATCCTCTAGTTCATGAACCAC	58.0	40	
MaMSD1A	MSD1A-ORFF: AAAGCTTGTGGGCGATCGGC	54.0	45 (for ORF),	ORF/gDNA amplification
	MSD1A-ORFR: TTGATACAGCAGAACAGTGACAAC		270 (for gDNA)	
	MSD1A-3RACEF1: GAAAGCCTTCACAACAGCTTCTGG	57.0	56	3′ RACE
	MSD1A-3RACEF2: CCCTTCTTGTTCCCCTGACTAG	58.0	12	
	MSD1A-5RACER1: TGCCCAGCAAAGGAACCAAGTTTG	57.0	48	5′ RACE
	MSD1A-5RACER2: GACGGAATCCAGAAGCTGTTGTGAAG	58.0	34	
MaMSD1B	MSD1B -ORFF: ATGGCGCTTCGAGCCCTCTTCAC	57.0	45 (for ORF),	ORF/gDNA amplification
	MSD1B -ORFR: TCAAGCGATCTCGCTGTCGTAAACTTCAC		180 (for gDNA)	
	MSD1B-3RACEF1:ACTCACTCGAAGCAGCTCTCAG	58.0	35	3′ RACE
	MSD1B-3RACEF2:ACAGGTGTTGCCATGGCGCTTC	61.0	23	
	MSD1B-5RACER1: GGTAGTATGCATGCTCCCACACATCG	60.0	45	5′ RACE
MaMSD1C	MSD1C-3RACEF1: CGTCACCAACTACAACAACGCCCTC	57.2	45	3′ RACE
	MSD1C-3RACEF2: CATATCAACCACTCGATCTTCTGG	53.7	45	
	MSD1C-5RACER1: CGACAGTGAGCTTCTTAAACCCCTTATC	56.4	42	5′ RACE
	MSD1C-5RACER2: GATTGTCCACCCAAGTGAACTGTGTG	58.0	42	
	MSD1C-ORFF: ATGGCTCTCCGGACCCTCTTC	56.4	42 (for ORF),	ORF/gDNA amplification
	MSD1C-ORFR:TCAGGCGGTCTCCTTCTCATAC		280 (for gDNA)	
MaMSD1D	MSD1D-ORFF1: ATGGCTCTCCGAACCCTCTTC	56.0	42 (for ORF),	ORF/gDNA amplification
	MSD1D-ORFR1: TTATGCAGTCTCTTTCTCATACACTTCAC		190 (for gDNA)	
	MSD1D-3RACEF1:GCCTTGGATTGGGTCTCGGC	57.0	48	3′ RACE

	MSD1C-3RACEF2: TCCACTGGTGACAAAAGGTTTGC	58.0	24	
	MSD1D-5RACER1: CACGACCGGGAAGAGAATGAAAGC	57.0	48	5′ RACE
	MSD1D-5RACER2: ACCAAGCAAAGGAACCATGTGCAAAC	58.0	42	
	FSD1A-ConrF: GARTWYCAYTGGGGRAAGCAYCA	57.0	30	Conserved region amplification
	FSD1A-ConrR: GTARTARGCRTGCTCCCAWACATC			
MaFSD1A	FSD1A-3RACEF1:CATCTGGAAGGCTTATGGAACTCATC	56.0	35	3′ RACE
	FSD1A-3RACEF2:CTGGTTGGGCTTGGCTTGTCTAC	60.0	30	
	FSD1A-5RACER1:GATAGTAAGCGTGCTCCCAGACATCG	60.0	45	5′ RACE
	FSD1A-ORFF1: ATGCAGCATCATACTCTTTCCTGTTG	55.0	55 (for ORF),	ORF/gDNA amplification
	FSD1A-ORFR:TCATTCAGCCTCCGAATCATCG		240 (for gDNA)	
	FSD1B-3RACEF1:GGTTGCACAATGGAGGAGCTTATC	56.0	57	3′ RACE
	FSD1B-3RACEF2:CTTCTGGGAATCAATGCAACCTAATGG	56.0	53	
MaFSD1B	FSD1B-5RACER1:CCATTAGGTTGCATTGATTCCCAGAAG	56.0	32	5′ RACE
	FSD1B-5RACER2: GATAAGCTCCTCCATTGTGCAACC	56.0	25	
	FSD1B-ORFF: ATCCGCCGTCGTCCTACGC	56.2	60 (for ORF),	ORF/gDNA amplification
	FSD1B-ORFR:GTCTCCATAAGATATAACTTTCGCTTC		250 (for gDNA)	
	AUAP: GGCCACGCGTCGACTAGTAC	-	-	Universal primer for 3′ RACE
	10UPM:CTAATACGACTCACTATAGGGCAAGCAGTGGTAT CAACGCAGAGTACATGGGG	-	-	Universal primer for 5′ RACE
	NUPM:AAGCAGTGGTATCAACGCAGAGT			
