# Supplementary material for: Genome-wide identification and characterization of the superoxide dismutase gene family in Musa acuminata cv. Tianbaojiao (AAA group)
Source: BMC Genomics. 2015 Oct 20;16:823. doi: 10.1186/s12864-015-2046-7 (PMC4615540; doi:10.1186/s12864-015-2046-7)
Supplement: Additional file 2: Table S2. — Oligonucleotide primers used for cloning the 5’- flanking regions of MaSOD genes. (PDF 102 kb) [file 12864_2015_2046_MOESM2_ESM.pdf]

Additional file 2: Table S2. Oligonucleotide primers used for cloning the 5′- flanking regions of *MaSOD* genes.

Gene name	Prime sequences （5′-3′）	Tm / °C	Promoter length / bp
MaCSD1A	CSD1A-proF: GACCACTCTACAAGACCGTCG<br>CSD1A-proR: GATCGCCACGCCCTTCAGATTG	57.5	2007
MaCSD1B	CSD1B-proF: CTTTGATATCGACGGTAACTATCGAGC<br>CSD1B-proR: CATCCTCACTGCCACCAAGAAC	58.0	2005
MaCSD1C	CSD1C-proF: CTCAATACAGTTGATCATTTGCATCAC<br>CSD1C-proR: GCCCTTGACGCTATCACTGCTAC	57.0	1565
MaCSD1D	CSD1D-proF: GCTTTCATTCACGCACTCCATTGC<br>CSD1D-proR: CAGCTACAGCCTTAACCATGTTC	56.4	2114
MaCSD2A	CSD2A-proF: TAGGGTCTTTGTTTGAAGGTGAATC<br>CSD2A-proR: TGTGGCTTGCATCAACCCGAAG	56.0	1986
MaMSD1A	MSD1A-proF: CAATTCATAGTACATAGGATCATGGC<br>MSD1A-proR: AATCCAGAAGCTGTTGTGAAGGC	55.0	2077
MaMSD1B	MSD1B-proF: AACTTTCTCAAATCCATTCCAATCGTC<br>MSD1B-proR: AAGCTAGGGTTGCGGCTGTCTTC	57.0	1977
MaMSD1C	MSD1C-proF: CTGATAAAAGGTGCATCTGTGTCAGC<br>MSD1C-proR: TTGGTGAAGAGGGTCCGGAGAG	58.0	2052
MaMSD1D	MSD1D-proF: CGTGAAGCATCTGCTCAAATGTTGC<br>MSD1D-proR: AGGCTCGAGTTAGGGCTTTCTTG	58.5	2044
MaFSD1A	FSD1A-proF: GGCACTACTGACGTGACGTGTG<br>FSD1A-proR: CAACAGGAAAGAGTATGATGCTGCA	58.0	1973
MaFSD1B	FSD1B-proF: CAACTCCATTTCACATTCAGCAGTAG<br>FSD1B-ORFR:GTCTCCATAAGATATAACTTTCGCTTC	57.0	1084
