# Supplementary material for: Genome-wide identification and characterization of the superoxide dismutase gene family in Musa acuminata cv. Tianbaojiao (AAA group)
Source: BMC Genomics. 2015 Oct 20;16:823. doi: 10.1186/s12864-015-2046-7 (PMC4615540; doi:10.1186/s12864-015-2046-7)
Supplement: Additional file 3: Table S3. — Specific primers used for quantitative real-time PCR. (PDF 62 kb) [file 12864_2015_2046_MOESM3_ESM.pdf]

Additional file 3: Table S3. Specific primers used for quantitative real-time PCR.

Gene name	Primer（5′-3′）	Amplicon / bp	Tm / °C
MaCSD1A	CSD1A-QF:CATATGCACGAACCACTGTC<br>CSD1A-QR: GGCTCATCATTACAAGAGGTCTA	140	57
MaCSD1B	CSD1B-QF:CTCCAAGGTTGAGCAAGTTG<br>CSD1B-QR: CTAATAAGGAAAAGGAAGCATAGG	129	57
MaCSD1C	CSD1C-QF:GCCGATCCAGATGATCTTG<br>CSD1C-QR: ACCTCACCATCCAACCAATAG	137	57
MaCSD1D	CSD1D-QF:TCAGGAGTGACTGATCCAAGG<br>CSD1D-QR: AGCCAAAGAAAAGAAGCCATC	150	57
MaCSD2A	CSD2A-QF:GAAATGCTGGAGGAAGATTGG<br>CSD2A-QR: CCATTAGGTTCATCGACTTGG	131	57
MaCSD2B	CSD2B-QF:TGCTTCTTTCACAGACGGTG<br>CSD2B-QR: AACAGGCTTATTAGGTGGCATC	175	57
MaMSD1A	MSD1A-QF:GAAGTTTTCGATGAAGAGACTGC<br>MSD1A-QR:TTGATACAGCAGAACAGTGACAAC	103	57
MaMSD1B	MSD1B-QF:TGCCAGTGAAGTTTATGACAGC<br>MSD1B-QR:CCAGGTGACATACTGATGGTG	156	57
MaMSD1C	MSD1C-QF:GTGAATTTTCCGGCATGTAA<br>MSD1C-QR:CAGGGCATGAACAAGTAGTGAC	148	57
MaMSD1D	MSD1D-QF:TTGCTTTCATTCTCTTCCCG<br>MSD1D-QR: CCTGGGGCATTGATAGATACTG	132	57
MaFSD1A	FSD1A-QF:CTCCAAGGTTGAGCAAGTTG<br>FSD1A-QR: CTAATAAGGAAAAGGAAGCATAGG	149	57
MaFSD1B	FSD1B-QF:CGTTACAAAGGTTCTGGCATACTA<br>FSD1B-QR:CAACCATACAATGGGCTGTTA	168	57
MaCAC	CAC-QF:AACTCCTATGTTGCTCGCTTATG<br>CAC-QR:GGCTACTACTTCGGTTCTTTCAC	148	57
