# Supplementary material for: Genome-wide identification and characterization of the superoxide dismutase gene family in Musa acuminata cv. Tianbaojiao (AAA group)
Source: BMC Genomics. 2015 Oct 20;16:823. doi: 10.1186/s12864-015-2046-7 (PMC4615540; doi:10.1186/s12864-015-2046-7)
Supplement: Additional file 4: Table S4. — Information of SOD genes from the wild and cultivated bananas. (PDF 13 kb) [file 12864_2015_2046_MOESM4_ESM.pdf]

Additional file 4: Table S4. Information of *SOD* genes from the wild and cultivated bananas.

Musa acuminata var. DH-Pahang (AA group)			*Musa balbisiana* var. PKW (BB group)			*Musa acuminata* cv. Tianbaojiao (AAA group)			
Locus ID	chr	ORF, bp	Locus ID	ORF, bp	Identity[c], %	Gene name	ORF, bp	Identity[d], %	Identity[e], %
GSMUA_Achr4T06850_001	4	483	ITC1587_Bchr4_T08984	597	65.56	*MaCSD1A*	483	98.76	63.47
GSMUA_Achr2T02380_001	2	459	ITC1587_Bchr2_T03311	5067[b1]	8.64	*MaCSD1B*	459	99.56	8.66
GSMUA_Achr7T03680_001	7	459	ITC1587_Bchr7_T18882	459	95.42	*MaCSD1C*	459	99.35	95.67
GSMUA_Achr8T09910_001	8	447[a]	ITC1587_Bchr8_T22308	3204[b2]	10.31	*MaCSD1D*	459	83.82	10.69
GSMUA_Achr9T20410_001	9	690	ITC1587_Bchr9_T27285	681	78.63	*MaCSD2A*	684	96.52	81.30
GSMUA_Achr10T06740_001	10	651	ITC1587_Bchr10_T29543	633	94.93	*MaCSD2B*	675	95.41	91.59
GSMUA_Achr8T21350_001	8	717	ITC1587_Bchr8_T23906	729	80.66	*MaMSD1A*	717	99.44	79.52
GSMUA_Achr3T18000_001	3	732	ITC1587_Bchr3_T07100	855	83.86	*MaMSD1B*	732	99.32	83.51
GSMUA_Achr2T18870_001	2	738	ITC1587_Bchr2_T04759	741	97.98	*MaMSD1C*	738	98.78	97.84
GSMUA_Achr3T09840_001	3	726	ITC1587_Bchr3_T06055	837	85.19	*MaMSD1D*	732	98.63	85.66
GSMUA_Achr11T23560_001	11	906	ITC1587_Bchr11_T34129	780	82.12	*MaFSD1A*	906	98.90	83.00
GSMUA_Achr10T27190_001	10	783	ITC1587_Bchr10_T31275	1491	28.09	*MaFSD1B*	783	99.74	28.15
GSMUA_Achr10T27220_001	10	783	ITC1587_Bchr10_T31280	1275	52.81			98.85	52.35

a: This gene is annotated as partial sequence in the 'DH-Pahang' genome database.

b1: A chimeric gene contains fragments of *Cu/ZnSOD* and dnaj heat shock domain.

b2: A chimeric gene contains fragments of *Cu/ZnSOD* and pentatricopeptide repeat-containing protein.

c: ORF identity of the *SOD* genes from the two wild bananas.

d: ORF identity of *MaSODs* and 'DH-Pahang' *SOD* genes.

e: ORF identity of *MaSOD*s and 'PKW' *SOD* genes.

Length differences of the ORFs among bananas are marked in colors.
