# Supplementary material for: Genome-wide identification and characterization of the superoxide dismutase gene family in Musa acuminata cv. Tianbaojiao (AAA group)
Source: BMC Genomics. 2015 Oct 20;16:823. doi: 10.1186/s12864-015-2046-7 (PMC4615540; doi:10.1186/s12864-015-2046-7)

Additional file 5: Figure S1. Sequence alignment of *MaFSD1B* and *MaFSD1B-variant1*. Start and termination codons are showed in red box. Two extra sequences are marked in mazarine.

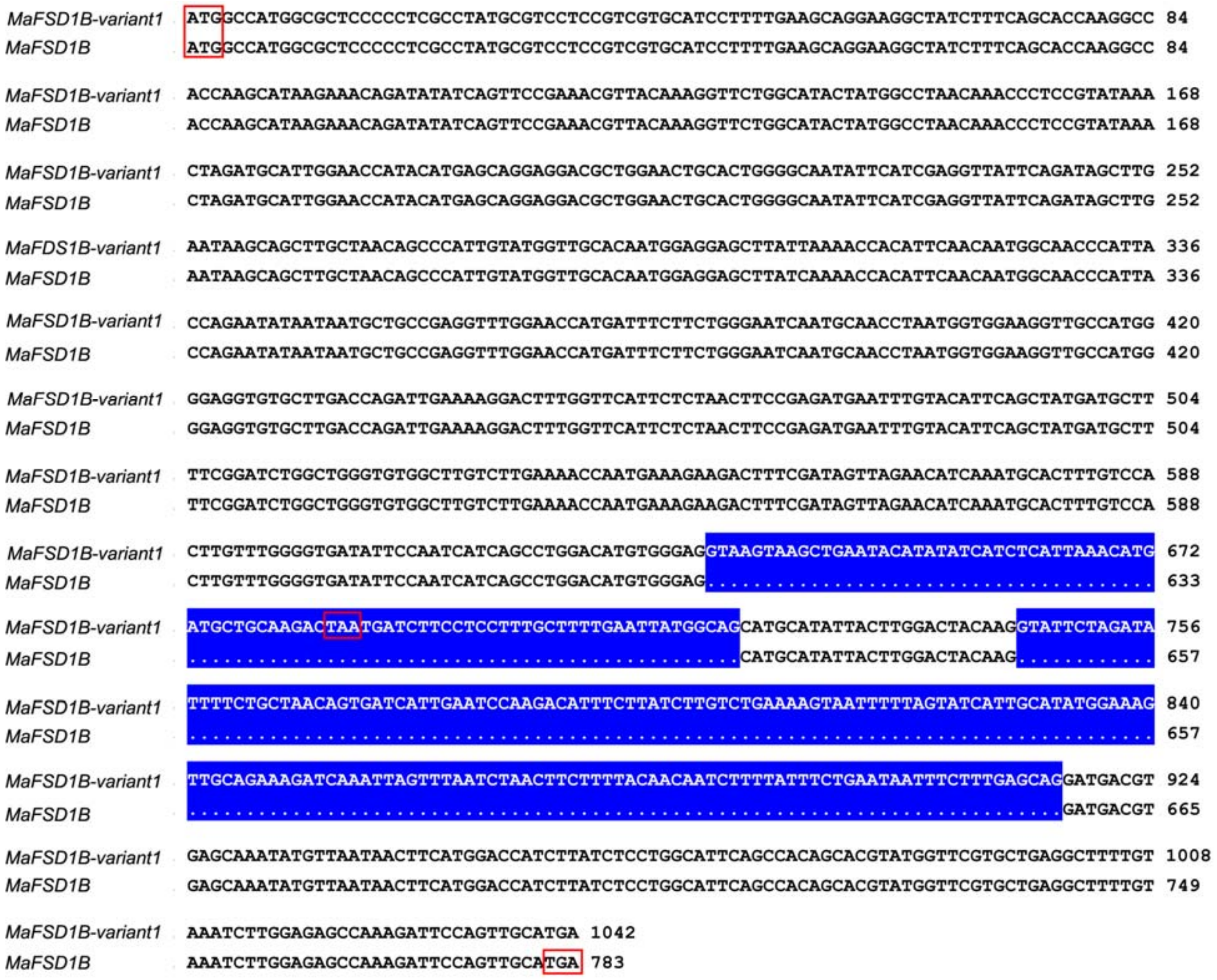

Supplement: Additional file 5: Figure S1. — Sequence alignment of MaFSD1B and MaFSD1B-variant1. (PDF 211 kb) [file 12864_2015_2046_MOESM5_ESM.pdf]
