# Supplementary material for: Genome-wide identification and characterization of the superoxide dismutase gene family in Musa acuminata cv. Tianbaojiao (AAA group)
Source: BMC Genomics. 2015 Oct 20;16:823. doi: 10.1186/s12864-015-2046-7 (PMC4615540; doi:10.1186/s12864-015-2046-7)
Supplement: Additional file 6: Table S5. — Pairwise identity of the coding region cDNAs and deduced amino acids of MaSOD genes. (PDF 13 kb) [file 12864_2015_2046_MOESM6_ESM.pdf]

Additional file 6: Table S5. Pairwise identity of the coding region cDNAs and deduced amino acids of *MaSOD* genes.

		Pairwise identity of nucleotide acid (%)											
		MaCSD1A-1	*MaCSD1B*	*MaCSD1C*	*MaCSD1D*	*MaCSD2A*	*MaCSD2B*	*MaMSD1A*	*MaMSD1B*	*MaMSD1C*	*MaMSD1D*	*MaFSD1A*	*MaFSD1B*
Pairwise identity of deduced amino acid (%)	MaCSD1A-1	-	57.4	58.8	57.4	42.0	43.2	22.3	21.0	23.5	20.8	18.1	20.3
	MaCSD1B	61.3	-	82.8	73.0	42.7	43.6	20.8	20.1	20.6	20.5	18.4	20.7
	MaCSD1C	60.6	87.5	-	71.5	42.3	42.8	21.5	23.3	23.3	22.2	18.0	18.9
	MaCSD1D	55.6	71.7	71.1	-	42.0	42.1	21.4	19.6	19.1	21.2	18.0	14.8
	MaCSD2A	39.7	42.3	41.6	40.1	-	83.7	32.5	29.6	33.0	29.1	27.1	20.2
	MaCSD2B	40.6	42.4	43.3	39.7	75.8	-	30.8	32.3	31.9	32.9	25.6	23.3
	MaMSD1A	9.9	8.7	9.9	11.8	15.7	15.0	-	80.9	84.8	85.0	31.3	33.8
	MaMSD1B	11.3	10.6	11.0	9.9	15.1	13.9	81.9	-	81.7	81.0	32.7	35.6
	MaMSD1C	12.1	9.1	11.3	10.1	15.0	13.0	86.1	82.0	-	85.9	33.6	28.9
	MaMSD1D	11.7	10.8	10.8	14.4	14.7	13.2	84.6	81.1	86.5	-	32.6	28.8
	MaFSD1A	8.4	6.6	6.3	8.5	10.6	11.9	22.7	18.1	24.0	23.4	-	42.5
	MaFSD1B	10.8	9.85	9.6	9.7	13.2	12.1	22.6	23.6	24.9	24.9	33.3	-

Pairwise identities of *MaSODs* in the same type are marked in colors.
