# Supplementary material for: Genome-wide identification and characterization of the superoxide dismutase gene family in Musa acuminata cv. Tianbaojiao (AAA group)
Source: BMC Genomics. 2015 Oct 20;16:823. doi: 10.1186/s12864-015-2046-7 (PMC4615540; doi:10.1186/s12864-015-2046-7)
Supplement: Additional file 7: Table S6. — Motif sequences of MaSODs identified by MEME tools. (PDF 14 kb) [file 12864_2015_2046_MOESM7_ESM.pdf]

Additional file 7:Table S6. Motif sequences of *MaSODs* identified by MEME tools. Cu/ZnSOD signatures (GFH[VL]H[EA][FL]GDTT and GNAG[GA]R[VL]ACG) are underline, Cu^{2+} binding sites and Zn^{2+} binding sites are in box and red box, respectively. The conserved metal-binding domain "DVWEHAYY" for MnSOD or FeSOD are in green box. Five residues that are present only in MnSOD and absent from FeSOD are in blue box. Residues important for distinguishing FeSOD from MnSOD are in grey.

Motif	Consensus sequence	Known motif description
Motif1	GPTTV[NT][GV]R[IV][ST]GL[KT]PGLHGFH[VL]H[EA][FL]GDTTNGC [MI]STG[PA]HFNPx[GK][KM][ET]HGAPEDEVRHAGDLGN[IV][VT]A [GN]EDG[TV][AV][ET][AF][TS][IK]VD[SN]QIPLSGPNS[IV][IV]GRA VVVH[AE][DL][PE]DDLG[KR]GGHELS[KL][TS]TGNAG[GA]R[VL]AC G[IV][IV]GL[QT]	Cu/ZnSOD
Motif2	KGD[AV][PST]AVVRLQ[SG]AIKFNGGGHINHSIFW[KN]NLKPV[NK]EG GGEPPH[SA][TAS]LGW[AT]IDTDF[GC]SLEALVQKMNAEGAALQGSGW VWLALDK[GE][SFR]KKL[CLST]VE[TA]TANQDPLVTKG[AL][NH][LM]V PLLGIDVWEHAYYLQYKNV[RK]PDYLKNIW[KDS]V[IM][ND]WKYASEV [YF][DE][KES]E	MnSOD
Motif3	[GF]LRQARGL[TA]VAALPD[LP]PYD[YL]GALEPA[IM]S[GR]E[IT][ML] [RE]LH[HW][QG][KR]HH[QR]AYVT[NS][YL]NNAL[EA]QL[ED]	
Motif4	[CM][GT][LM][DE]E[IL][IV][KV][TV][ST][FY]N[KN]GNPLP[EL][FY] [NV][HN]AA[EQ]VWNHDFFW[EQ]S[IM][KQ]P[GN]GG[GR][LR]P[SW] G[GR][LV][LM][DE][HQ]IE[KR]DFGSF[DS][NR][FM][LR][DE]EF[KV] [HR][AS]A[LM][MT][LQ]FGSGW[AV]WLV[LY]K[AT]N	
Motif5	AV[AV]VL[GK][GS][NST][ESD][DSG]V[EKG]G[VT][VI][YT][FL][SVA]QE	
Motif6	M[AS]LR[TA]LF[TS]K[KT][AS][LAF][TA][LRT]AS[GA][FL][GR]P	Mitochondrial location signal
Motif7	[CN]P[IL]VW[DG][DY][IS]P[IL][IL][AS][IL]D[MV]WEHAYYLDY [EK][DN][DR][RV][AS][DK]YV[NS][IN]F[LM][DE][HK]L[IV]SW	
Motif8	MQA[IT]LAAAM[AP]A[HQ]T[LV]L[FL]SASS	Chloroplast location signal
Motif9	[MS]A[LR]PL[AL][RY][AS][AS][LS][LV][GV][HQ]PF[ER][AC][GL][PR][FL]S F	Chloroplast location signal

Significant motifs (p-value<1e-100) of more than 20 amino acid length are predicted by MEME search.
