# Supplementary material for: Genome-wide identification and characterization of the superoxide dismutase gene family in Musa acuminata cv. Tianbaojiao (AAA group)
Source: BMC Genomics. 2015 Oct 20;16:823. doi: 10.1186/s12864-015-2046-7 (PMC4615540; doi:10.1186/s12864-015-2046-7)
Supplement: Additional file 8: Table S7. — Potential functional SOD family genes identified in silico in monocot plants. (PDF 13 kb) [file 12864_2015_2046_MOESM8_ESM.pdf]

Additional file 8: Table S7. Potential functional *SOD* family genes identified *in silico* in monocot plants.

Species name	*Cu/ZnSOD* genes		*MnSOD* genes		*FeSOD* genes		Websites for genome sequences downloading[a]
	Locus ID	Total NO.	Locus ID	Total NO.	Locus ID	Total NO.	
Oryza sativa (ssp. japonica)	LOC_Os03g22810<br>LOC_Os07g46990<br>LOC_Os08g44770<br>LOC_Os03g11960	4	LOC_Os05g25850	1	LOC_Os06g02500<br>LOC_Os06g05110	2	http://phytozome.jgi.doe.gov/pz/portal.html#!info?alias=Org_Osativa
Zea mays	GRMZM2G025992<br>GRMZM2G058522<br>GRMZM2G106928<br>GRMZM2G169890<br>GRMZM5G891739	5	GRMZM2G059991<br>GRMZM2G124455	2	GRMZM5G864424<br>GRMZM2G042080<br>GRMZM2G173628	3	http://phytozome.jgi.doe.gov/pz/portal.html#!info?alias=Org_Zmays
Brachypodium distachyon	Bradi1g18340<br>Bradi1g69680<br>Bradi3g43070	3	Bradi2g30580	1	Bradi1g50550<br>Bradi1g51140	2	http://phytozome.jgi.doe.gov/pz/portal.html#!info?alias=Org_Bdistachyon
Sorghum bicolor	Sobic.001G371900<br>Sobic.007G166600<br>Sobic.002G407900<br>Sobic.001G453800	4	Sobic.009G093200	1	Sobic.010G012900<br>Sobic.010G033000	2	http://phytozome.jgi.doe.gov/pz/portal.html#!info?alias=Org_Sbicolor
Hordeum vulgare	MLOC_22278<br>MLOC_38479<br>MLOC_17760	3	MLOC_72880	1	MLOC_69817<br>MLOC_36735	2	http://plants.ensembl.org/Hordeum_vulgare/Info/Index/
Triticum aestivum	Traes_4AS_8AC85AB54<br>Traes_4BL_F765C4AA5<br>Traes_4DL_08EEED1A6<br>Traes_2AS_529825553<br>Traes_2BS_6015BC7C6	10	Traes_2AL_D0D84176E<br>Traes_2DL_F48387F4E	2	Traes_7AS_ECBB6F731<br>Traes_4AL_433F090E0<br>Traes_7DS_1CE2F67B7<br>Traes_7AS_8400350BF<br>Traes_4AL_7742FFD9E	6	http://phytozome.jgi.doe.gov/pz/portal.html#!info?alias=Org_Taestivum_er

	Traes_2DS_3C3A2A12A<br>Traes_7BL_70F07C889<br>Traes_7AL_EAE52A86A<br>Traes_7BL_A42D6C984<br>Traes_7AL_E14A72218				Traes_7DS_B5FBEEE92		
Setaria italica	Si031388m<br>Si037512m<br>Si014166m<br>Si037598m	4	Si023127m	1	Si038762m<br>Si007118m	2	http://phytozome.jgi.doe.gov/pz/portal.html#!info?alias=Org_Sitalica
Phalaenopsis equestris	PEQU_04990<br>PEQU_28621<br>PEQU_03550<br>PEQU_42061<br>PEQU_00184	5	PEQU_18674	1	PEQU_20491<br>PEQU_37345<br>PEQU_39485	3	ftp://ftp.genomics.org.cn/from_BGISZ/20130120/02.annotation/

a: All peptide sequences of sequenced monocots were download from the related genome websites, and the *Arabidopsis* and banana *SOD* protein sequences were used as queries to perform local BLASTp searches to identify *SOD* gene members. We then confirmed all the protein sequences by BLASTp searches in the NCBI for the presences of the conserved SOD domains. Redundant genes, chimeric genes and genes with a very small SOD domain were excluded.
